# Free health care for under-fives, expectant and recent mothers? Evaluating the impact of Sierra Leone’s free health care initiative

**DOI:** 10.1186/s13561-016-0096-4

**Published:** 2016-05-23

**Authors:** Ijeoma Edoka, Tim Ensor, Barbara McPake, Rogers Amara, Fu-Min Tseng, Joseph Edem-Hotah

**Affiliations:** 1 PRICELESS, Wits School of Public Health, Johannesburg, South Africa; 2 Institute of Health Sciences, University of Leeds, Leeds, UK; 3 Institute for Global Health and Development, Queen Margaret University Edinburgh, Edinburgh, UK; 4 Nossal Institute for Global Health, University of Melbourne, Melbourne, Australia; 5 Rebuild Consortium, College of Medicine and Allied Health Sciences, University of Sierra Leone, Freetown, Sierra Leone; 6 Department of Surgery and Cancer, Imperial College London, London, UK

## Abstract

This study evaluates the impact of Sierra Leone’s 2010 Free Health Care Initiative (FHCI). It uses two nationally representative surveys to identify the impact of the policy on utilisation of maternal care services by pregnant women and recent mothers as well as the impact on curative health care services and out-of-pocket payments for consultation and prescription in children under the age of 5 years. A Regression Discontinuity Design (RDD) is applied in the case of young children and a before-after estimation approach, adjusted for time trends in the case of expectant and recent mothers. Our results suggest that children affected by the FHCI have a lower probability of incurring any health expenditure in public, non-governmental and missionary health facilities. However, a proportion of eligible children are observed to incur some health expenditure in participating facilities with no impact of the policy on the level of out-of-pocket health expenditure. Similarly, no impact is observed with the utilisation of services in these facilities. Utilisation of informal care is observed to be higher among non-eligible children while in expectant and recent mothers, we find substantial but possibly transient increases in the use of key maternal health care services in public facilities following the implementation of the FHCI. The diminishing impact on utilisation mirrors experience in other countries that have implemented free health care initiatives and demonstrates the need for greater domestic and international efforts to ensure that resources are sufficient to meet increasing demand and monitor the long run impact of these policies.

## Background

The destruction of health infrastructure and flight of qualified health professionals following 11 years (1991–2002) of brutal civil war contributed to the collapse of Sierra Leone’s health care system. Although some progress has been made in rebuilding the health care system, Sierra Leone has one of the world’s highest child mortality rates and maternal mortality ratios [1]. Characterised by weak infrastructure, shortage of equipment, medical supplies and qualified health professionals, the health care system lacks the capacity to provide adequate quality of care. Furthermore, high costs of care and the fear of incurring such costs deter the use of health care services when needed [2].

Against this backdrop, the Free Health Care Initiative (FHCI) was launched on 29^th^ April 2010 to increase access to formal health care services and address poor maternal and child health outcomes [3, 4]. Under the FHCI, children under the age of 5 years, pregnant women and mothers of young babies (lactating) are exempted from paying any medical charges including consultation fees, medicines and medical supplies in all government health care facilities as well as in facilities contracted by the government to provide free health care under the initiative. Unlike pre-existing national health care exemption rules, the FHCI received significant financial backing from the Sierra Leone Government and international donor agencies [5, 6]. Furthermore, the FHCI was accompanied by a range of health sector reforms to improve efficiency in the delivery of health care services. These reforms, which include strengthening the procurement and supply chain management system, strengthening and redistribution of the health workforce as well as payroll cleansing, were to ensure the adequate flow of medical supplies and equipment to points of need as well as the supply of trained health workers particularly to rural areas experiencing critical shortages [3, 7].

In the first few months following the implementation of the FHCI, utilisation of health service increased sharply amongst all beneficiary groups [4, 8]. For example, use of health care services by children under 5 years increased 2.5-fold, the number of pregnant women making at least one antenatal care (ANC) visit increased by 20 % and average monthly institutional deliveries increased by approximately 18 % [4]. In another study, Groen, Kamara [9] reported a 500 % increase in the number of children under 5 years receiving surgical care, which was considerably larger than the 17 % increase observed in children over 5 years.

Studies on the impact of the FHCI are limited and have either been descriptive in design or restrictive in sample sizes, thus limiting the strength of the findings. This study attempts to quantify the impact of the FHCI on child and maternal health care service-use and on out-of-pocket expenditure using large scale national data. For children, we use a Regression Discontinuity Design (RDD) and for maternal health care, a time trend-adjusted before-after estimation approach is applied. Our results suggest that while children under the age of five are less likely to incur out-of-pocket health expenditure, particularly consultation fees, no difference is observed in the absolute level of out-of-pocket expenditure incurred as well as in the use of curative health care services in facilities where the FHCI applies. For key maternal health services we find substantial, significant but possibly transient increases after the implementation of the FHCI.

The rest of the paper is structured as follows: the Methods section provides a description of the survey data used, key study variables and an outline of the estimation strategies; the results are presented and discussed in the third and fourth sections, respectively and the fifth section concludes the paper.

## Methods

### Data and variable description

The study makes use of two recent household surveys—the 2011 Sierra Leone Integrated Household Survey (SLIHS), to estimate the effect of the FHCI in children under the age of 5 years and the 2013 Sierra Leone Demographic and Health Survey (DHS), to estimate the effect on maternal health care seeking behaviours.

#### Sierra Leone Integrated Household Survey (SLIHS)

The 2011 SLIHS is a nationally representative sample of households in Sierra Leone. Approximately 6800 households were selected using a two-stage sampling process [10] and interviewed between January and December 2011. Data were collected on a wide range of individual and household characteristics, including health service use and health care expenditure. Utilisation of curative health services and out-of-pocket expenditure were obtained from the health module questionnaire completed by household heads or household representative persons for each household member. For utilisation, respondents are asked to list two out-patient facilities and health worker cadre visited in a 2-week period preceding the interview. Data on health expenditure and the owner of health facility (for example government/private/non-governmental organisation (NGO)/missionary-owned) were collected only for the first visit. Therefore, we define utilisation as the first out-patient health care facility visited when sick. Given that the FHCI applies only to public facilities as well as contracted NGOs and missionary health facilities, we estimate the effect of the FHCI in a subsample of those utilising these facilities. As a robustness check, a subsample of those utilising formal private and informal^1^ health facilities is used. Total out-of-pocket health expenditure^2^ is estimated as the sum of consultation and prescription expenditure incurred for the first out-patient visit. The final sample consists of children between 0 and 10 years who reported being ‘sick’ in the past 2 weeks and had complete data on health service utilisation, health expenditure, individual and household characteristics.

#### Demographic and Health Survey (DHS)

The most recent Sierra Leone DHS was conducted between June and December 2013 and interviewed 16,658 women of reproductive age (15–49). It collected detailed experiences of women relating to their most recent child birth over a 5-year recall period. This includes place of delivery, ANC and postnatal care, staff attending, delivery complications, content of ANC and vaccinations given to the baby during its first year. The 5-year retrospective nature of the survey means that information exists on births occurring both before and after the FHCI was introduced. Mothers’ and households’ socio-demographic characteristics are available in the 2013 DHS and used as controls. These include mothers’ age, education (no formal education/ primary/secondary/higher education) and religion (Christian/others); household location (rural/urban and region—Western, Eastern, Northern and Southern) and, household asset index quintile (lowest/second/third/fourth/highest).

### Estimation strategy

#### The Regression Discontinuity Design

The Regression Discontinuity Design (RDD) is used to identify the effect of the FHCI in children under the age of 5 years. The RDD is favoured over other quasi-experimental approaches for two reasons. First, RDD is applicable to a single cross-sectional survey when information on multiple time points is not available. Although an earlier survey was conducted in 2003, differences in survey design and data collection methodologies meant that data from the 2003 SLIHS were not always comparable to 2011. For example in 2003, data were not collected on prescription expenditure. Second, while a dummy variable approach^3^ can be applied to a single cross-sectional survey data, the fundamental difference between children between the age of 0–4 years (treatment group) and 5–10 years (control group) could result in biased estimate of the treatment effect. The RDD allows the use of one cross-sectional survey data while accounting for bias arising from potentially incomparable groups. It exploits the local randomisation of children around the eligibility threshold of the FHCI to identify the treatment effect. This implies that the level of treatment is discontinuous at a cut-off point or threshold value of the assignment variable (here, when age is greater or equal to 5 years). As a result of exposure to treatment, a causal effect can be inferred under the following identification assumptions [11, 12]: the change in the level of treatment at the eligibility threshold is truly discontinuous and the assignment variable is observed without error; in the absence of the treatment, the outcome of interest is a continuous function of the assignment variable at the eligibility threshold; and the eligibility threshold is exogenously determined. The last identification assumption which rules out sorting of individuals around the eligibility threshold assumes that individuals are randomly assigned around the locale of the threshold, resulting in a local randomized experiment [11, 12]. In other words, children just below the eligibility threshold are considered to be comparable to children just above the threshold. In this study the presence of a discontinuity in utilisation and out-of-pocket health expenditure at the eligibility threshold is tested using parametric regression analysis [12, 13].

Under the FHCI, all children under the age of five should automatically be eligible for free health care. However, due to limited compliance in some health facilities, not all eligible children receive free health care [14, 15]. Furthermore, some non-eligible children would have continued to receive free health care under pre-FHCI exemptions guidelines. Therefore, the receipt of free health care is not strictly determined by the eligibility rule. Under this scenario, the fuzzy RDD can be used as opposed to the sharp RDD [12].

The fuzzy RDD can be estimated using the instrumental variable approach:

1 $$ {O}_i = {\tau}_0+{\tau}_1{F}_i + f\left({Z}_i\right) + {e}_i $$

2 $$ {F}_i = {\alpha}_0 + {\alpha}_1{T}_i + g\ \left({Z}_i\right) + {\epsilon}_i $$

wheres *O*_*i*_ represents the study outcomes for child *i*, including utilisation and out-of-pocket health expenditure; *F* is a binary treatment indicator which equals 1 when child *i* receives free health care and zero otherwise; *Z*_*i*_ is age in months, rescaled to zero at the cut-off point (*i. e. Z*_*i*_ = *Aɡe*_*i*_ − 60); *T*_*i*_ is the eligibility indicator which takes the value of 1 if child *i*’s age is greater than or equal to 5 years and 0, otherwise (*T*_*i*_ = 1 {*Z*_*i*_ ≥ 5 years}); *f (Z*_*i*_*)* and *ɡ (Z*_*i*_*)* are polynomial functions representing the relationship between the rescaled age variable and the outcome variables; and *e*_*i*_ and s*ϵ*_*i*_ are random error terms.

In this study, *F* is not directly observed as data were not collected on the receipt of free health care. Therefore we estimate an intention-to-treat (ITT) effect, *τ*_*3*_ (equation 3), by substituting equation (2) into (1) to yield the reduced form [12]:

3 $$ {O}_i = {\tau}_0^r+{\tau}_3{T}_i + {f}^r\left({Z}_i\right) + {e}_i^r $$

where *τ*_*3*_ captures the difference between the outcomes of children above and below the eligibility threshold. A linear probability model is applied to estimate the effect on health service utilisation (and the probability of receiving free^4^ health care services). For total health care expenditure, a two-part model [16] is applied.

For each outcome, three models are specified to test the robustness of the results to different functional forms of *f (Z*_*i*_*)* : a simple linear specification, a linear interaction specification and a quadratic linear specification. The quadratic specification allows for non-linear relationships between (rescaled) age and the outcome variables, while the interaction specifications allows this relationship (or slope) to vary at either side of the eligibility threshold. The model that best fits the data or the optimal model specification is determined using the Akaike Information Criteria (AIC)^5^ [12].

Finally, the differential effect of the FHCI is estimated by disaggregating the full sample into subsamples defined by household location (rural/urban) and socioeconomic status (below and above median total household expenditure).

#### Time Trend-adjusted Before-After Estimation Approach

The FHCI was introduced throughout the country in late April 2010. As a result, there are no suitable control groups unaffected by the policy which can be used to compare the behaviour of the treatment group. We use a before-after design controlling for time trends to assess the impact of the FHCI on the utilisation of maternal health care services.

Four key behaviours are examined as binary outcome variables: obtaining four or more ANC visits (the WHO minimum recommendation during pregnancy) at a public facility (ANC4); delivery in a public facility and with a skilled health worker; postnatal care obtained within 48 h within a public facility together with vitamin A supplementation 2 months after birth; and uptake of child vaccination during the first year of birth (diphtheria, pertussis, tetanus plus hepatitis and haemophilus influenza type B given as part of a three course combined vaccination).

For delivery, postnatal care and vaccinations, we assume that births from May 2010 are affected by the launch of the FHCI. For antenatal outcomes, it is assumed that births after January 2011 are affected by the policy. The end date for maternal care was taken to be the month prior to the date of interview. For vaccinations the end date was taken as 1 year prior to the interview to ensure that adequate time had been given to provide the full course of inoculations.

First, we use a mixed effects multilevel logit model [17] unadjusted for time trends (Model 1) to estimate changes in maternal outcomes:

4 $$ {O}_{ijm}={\phi}_0 + {\phi}_2{P}_m + \varnothing {X}_{ijm} + {e}_j+{v}_m + {u}_{ijm} $$

Where *O*_*ijm*_ are the study outcomes for individual *i* in district *j* at month *m*; *P*_*m*_ is a policy variable which that takes a value of 1 when outcomes occur after the FHCI was implemented and 0 otherwise; *X*_*ijm*_ is a vector of covariates including asset index quintiles, age of mother, maternal education and whether location of birth was rural or urban, *e*_*j*_ and *v*_*m*_ are district and time specific random effects and *u*_*ijm*_ is an individual random error.

A weakness of the before and after design is that, in the absence of a counterfactual, there is no way of knowing whether the outcomes of interest would have changed in the absence of the FHCI. Although there is no comparison group, we mitigate this problem by incorporating a monthly time trend variable (*M*_*m*_) (equation 5) and investigating whether the trend changes after policy implementation. The time trend, *M*_*m*_, is coded from 1 (July 2008) to 61 (July 2013). Interaction between the month and policy variables (*M*_m *_P_*m*_) is added to allow for a different trend effect following the implementation of FHCI. The revised model (Model 2) becomes:

5 $$ {O}_{ijm}={\gamma}_0+{\gamma}_1{M}_m + {\gamma}_2{P}_m + {\gamma}_3{M}_m\ast {P}_m+\Lambda {X}_{ijm} + {\varepsilon}_j+{\eta}_m + {\mu}_{ijm} $$

Where, *ε*_*j*_ and $\eta$_*m*_ are district and time specific random effects and *μ*_*ijm*_ is an individual random error. We use a two-step test for plausible causality to examine whether the structural break in the data occurs after policy implementation. Rolling time-series estimation of average monthly outcomes for the entire sample period was obtained. Since average outcomes are proportions and so bounded between zero and one, a tobit model is specified using the rolling Stata function (window = 20). The resulting regression coefficients can then be graphed to visually identify structural breaks. A Stata function (estat sbsingle) is then also used to identify an unknown structural break date in the data. Any identified break in the data can in turn be used as an additional breakpoint for testing the effect of policy as well as acting as test of plausible causality since a break occurring before or distant from policy implementation suggests that some other factor is causing the change.

## Results

### Impact on utilisation and out-of-pocket health expenditure in children under 5 years

An important assumption of the RDD is that sorting of individuals around the eligibility threshold is completely random [12]. While this assumption cannot be directly tested, it implies similar distribution of individual characteristics on both sides of the eligibility threshold. Table 1 show that the two study groups appear balanced for the majority of observed characteristics except for two characteristics—age of household head and proportion of household heads with no formal education—both are higher in the group of children over the age of 5 years.

**Table 1:** Descriptive statistics (if sick)

	(1)	(2)	(3)	(4)
	Eligible children	Non-eligible children	Difference (2) – (1)	All study children
Child Characteristics				
Age in years	2.264	7.355	5.09^***^	4.944
Male	0.544	0.525	−0.019	0.534
Household Characteristics				
Rural	0.644	0.683	0.039	0.665
Western region	0.123	0.0948	−0.028	0.108
Eastern region	0.128	0.136	0.008	0.132
Northern region	0.422	0.474	0.052^*^	0.449
Southern region	0.327	0.296	−0.032	0.311
Household size	6.921	7.113	0.192	7.022
Household total expenditure per capita (US$)	293.8	276.2	−17.60	284.6
Head of Household Characteristics				
Married	0.851	0.865	0.014	0.859
Age	43.22	45.33	2.11^***^	44.33
Male	0.759	0.748	−0.01	0.753
Christian	0.175	0.157	−0.017	0.165
No education	0.693	0.752	0.059^**^	0.724
Observations	761	872		1633

^*^ *p* < 0.1, ^**^ *p* < 0.05, ^***^ *p* < 0.01

**Fig. 1:**
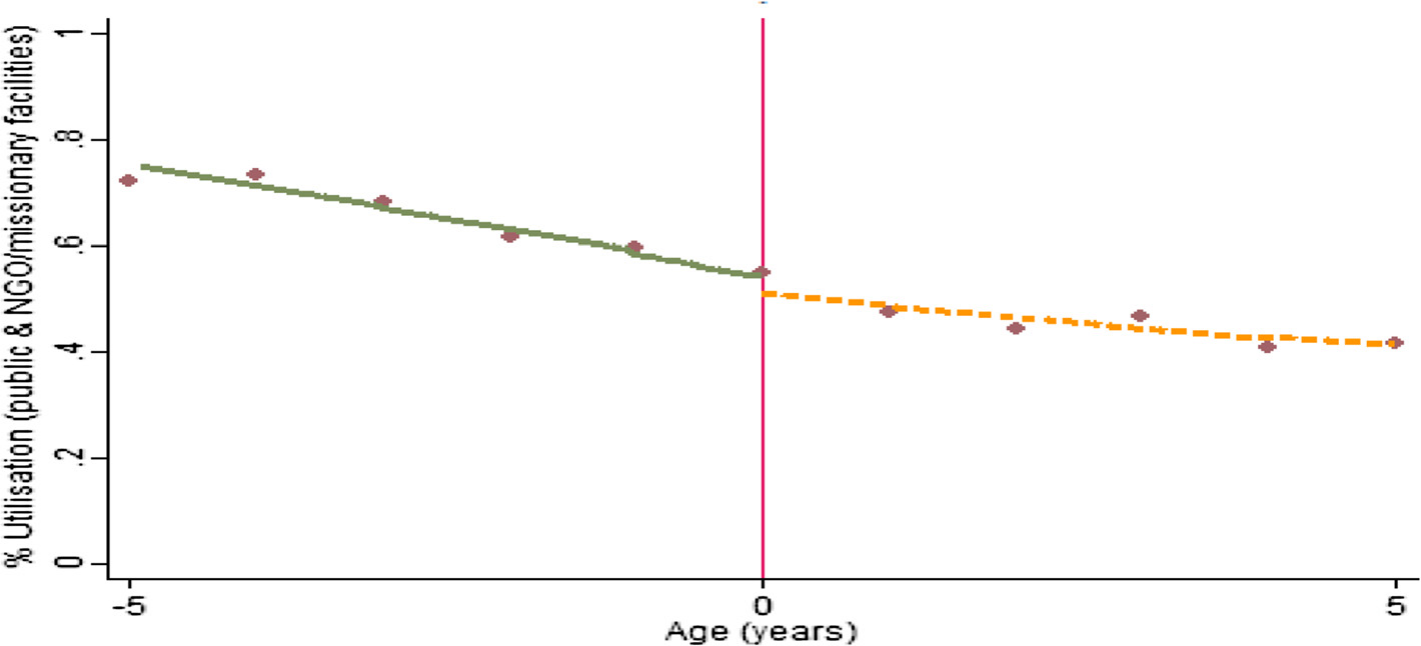
Non-parametric regression plot showing utilisation of public health care services below and above the eligibility threshold

**Fig. 2:**
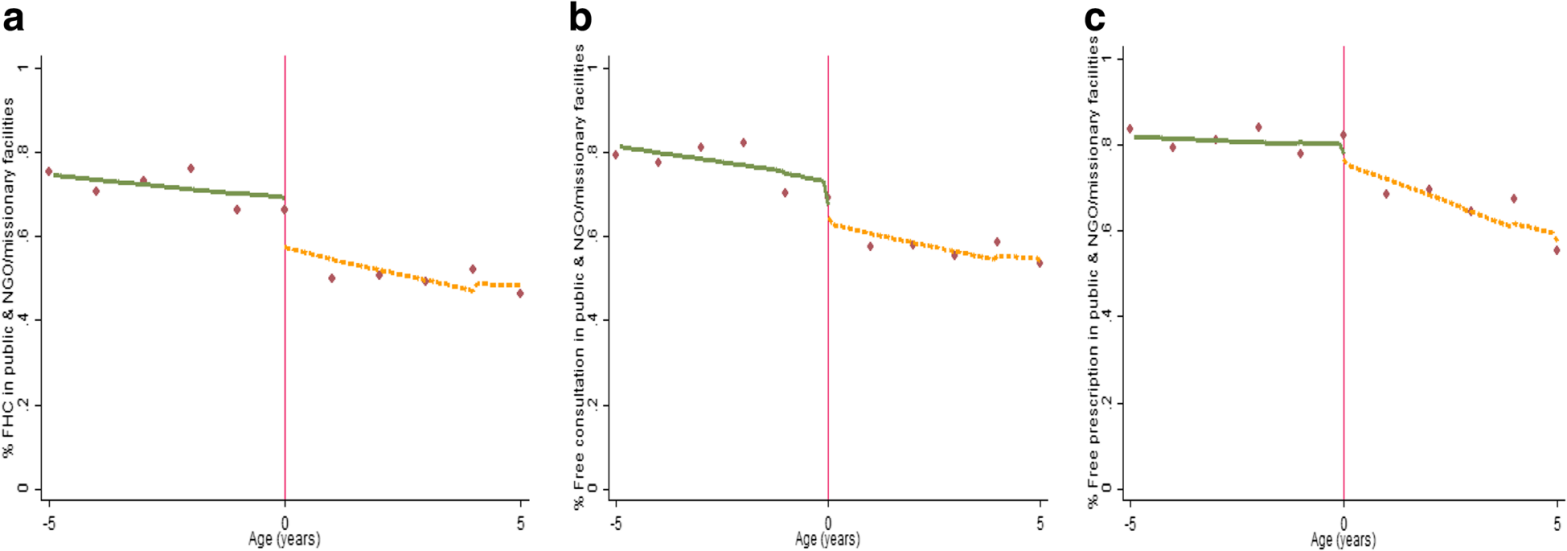
Non-parametric regression plots showing the probability of reporting zero health expenditure (for those who visit a public health facility), below and above the eligibility threshold (**a**): Zero consultation and prescription expenditure (**b**): Zero consultation expenditure (**c**): Zero prescription expenditure

**Fig. 3:**
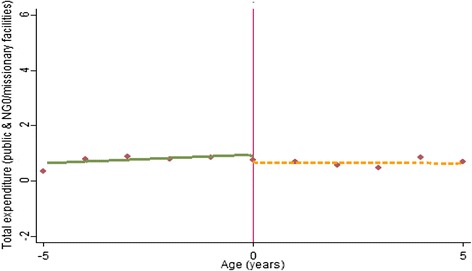
Non-parametric regression plot showing level of total out-of-pocket expenditure (US$) for expenditure greater than zero in public health facilities

First, we present a series of graphs (Figs. 1, 2, 3) depicting the relationship between the study outcome variables and (rescaled age). The graphs are estimated as local linear regression models on both sides of the eligibility threshold, using a triangle kernel and the default bandwidth from Imbens and Kalyanaraman [18]. A negative relationship is observed between age and use of public, NGO and missionary health care services (Fig. 1). However, only a small discontinuity is observed at the eligibility threshold where children just under the age of 5 years have a marginally higher probability of using public health care services (Fig. 1). A more distinct jump is observed with the proportions of children reporting zero health expenditure—children just under the age of 5 years are more likely to report zero consultation and prescription expenditure (Fig. 2a). This effect appears to be driven by a higher likelihood of reporting zero consultations (Fig. 2b), as opposed to prescription expenditure where no discontinuity is observed (Fig. 2c). However, for children with a positive expenditure, no clear discontinuity is observed (Fig. 3).

To provide statistical inferences on the presence of discontinuities at the eligibility threshold, we estimate a series of parametric models (Table 2). For each outcome, three models are specified—simple linear, linear interaction and quadratic interaction, and the optimal model specification identified on the basis of the lowest AIC. The result shows similar trends seen in Figs. 1, 2, 3. For example, at the optimal model specification (linear interaction), non-eligible children are less likely to report zero consultation and prescription expenditure. This appears to be driven largely by the lower likelihood of reporting zero consultation expenditure (at *p* > 0.1 in the optimal model specification). For those making positive payments, no statistically significant difference is observed with the level of total health expenditure. Similar to the full sample, the FHCI effect is also observed in children from more deprived backgrounds (children living in rural areas and in poorer households, Appendix 1: Table 6 and Appendix 2: Table 7 respectively). However, these effects are only significant at a 10 % level in the optimal model specification.

**Table 2:** The impact of the FHCI in public/NGO/missionary health facilities

	Simple linear	Linear interaction	Quadratic interaction
Utilisation (LPM)			
ITT effect (Age ≥ 5 years)	−0.0310	−0.0197	0.0885
	(0.060)	(0.061)	(0.098)
*N*	1633	1633	1633
*AIC*	2235.2	2236.5	2234.3^a^
Probability of reporting zero consultation and prescription expenditure (LPM)			
ITT effect (Age ≥ 5 years)	−0.134^*^	−0.142^**^	−0.0938
	(0.071)	(0.071)	(0.116)
*N*	915	915	915
*AIC*	1180.8^a^	1182.3	1186.0
Probability of reporting zero consultation expenditure (LPM)			
ITT effect (Age ≥ 5 years)	−0.121^*^	−0.126^*^	−0.0359
	(0.073)	(0.072)	(0.113)
*N*	915	915	915
*AIC*	1136.5^a^	1138.3	1141.2
Probability of reporting zero prescription expenditure (LPM)			
ITT effect (Age ≥ 5 years)	−0.0410	−0.0628	−0.0600
	(0.058)	(0.059)	(0.095)
*N*	915	915	915
*AIC*	1005.8	1003.9^a^	1007.8
Total expenditure (two-part model showing marginal effects)			
ITT effect (Age ≥ 5 years)	−0.144	−0.0827	−0.623
	(0.462)	(0.436)	(1.753)
*N*	915	915	915
*AIC*	686.0	685.94	685.93^a^

Control variables includes region dummies, head of households’ age and education

Standard errors in parentheses

*LPM* linear probability model

^*^ *p* < 0.1, ^**^ *p* < 0.05

^a^Optimal model specification

The only departure from the Figures is observed with utilisation of health care services where a positive ITT effect (higher utilisation in the non-eligible) is observed in the optimal model specification (Table 2). However, this effect is not statistically significant.

### Impact on formal private and informal/other health care service use

**Fig. 4:**
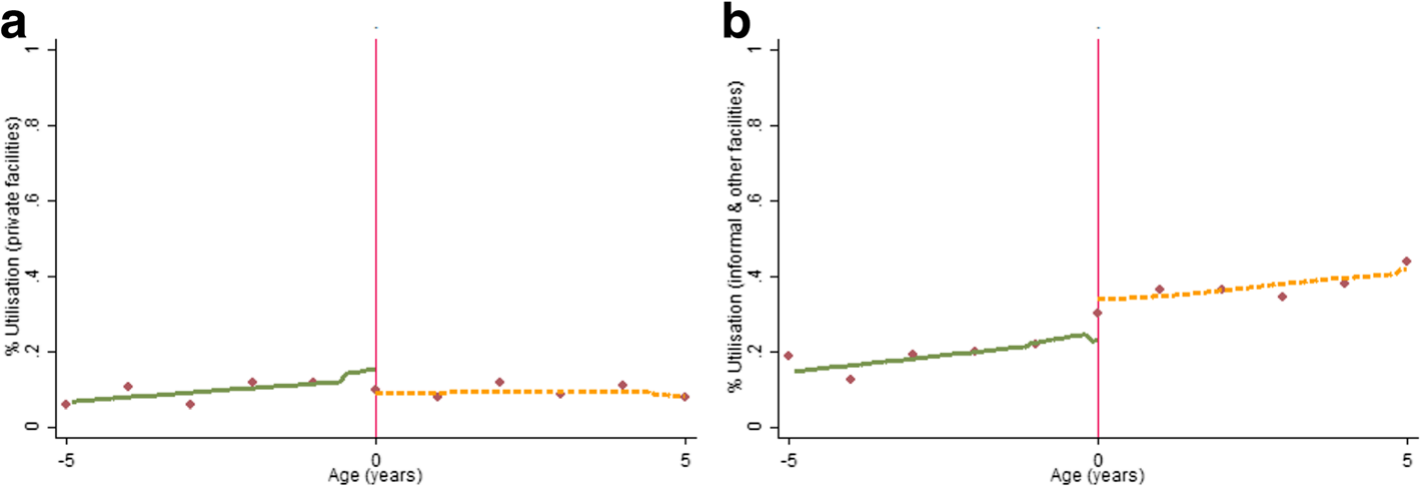
Non-parametric regression plots showing utilisation of health care services below and above the eligibility threshold, in: **a** Private formal health facilities **b** Informal/other health facilities

**Fig. 5:**
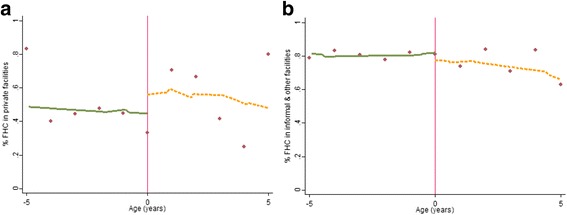
Non-parametric regression plots showing the probability of reporting zero health expenditure (for those who visit a health facility), below and above the eligibility threshold **a** Private formal health facilities **b** Informal/other health facilities

**Fig. 6:**
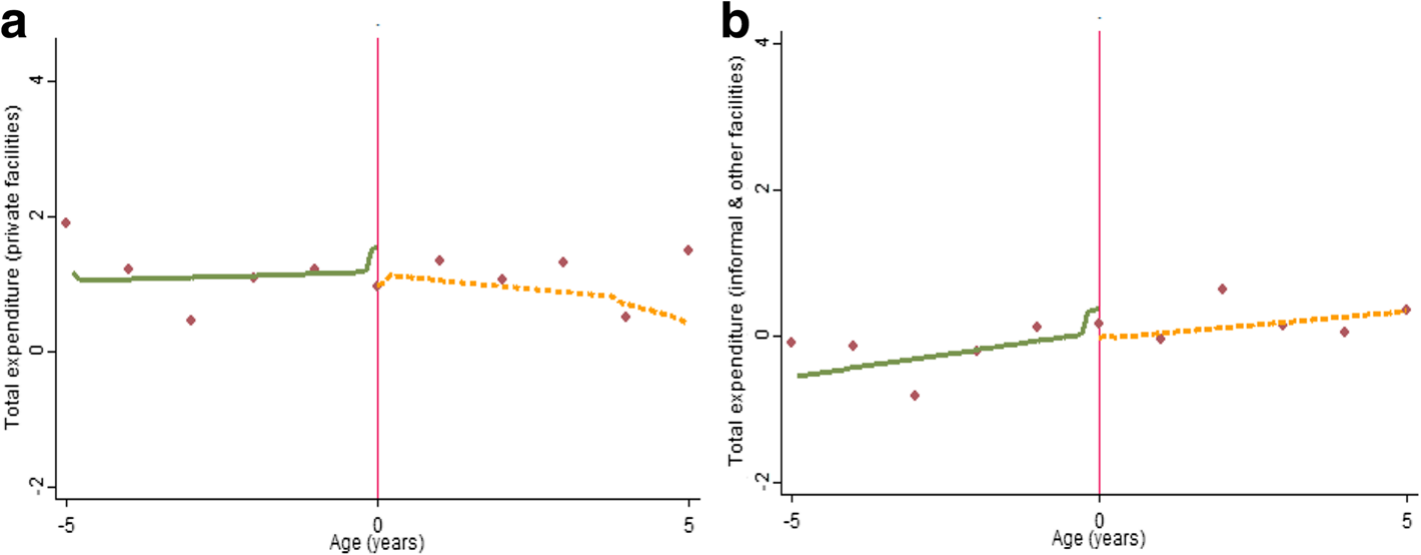
Non-parametric regression plots showing level of total out-of-pocket health expenditure (US$), for expenditure greater than zero **a** Private formal health facilities **b** Informal/other health facilities

Figures 4, 5, 6,^6^ graphically depict the effect of the FHCI on private and informal/other health care service use and out-of-pocket health expenditure. A distinct jump is observed at the eligibility threshold for informal service use—children over the age of 5 years are more likely to use these services (Fig. 4b). The result is confirmed by the parametric model which shows a statistically significant difference (of approximately 10 percentage points) (Table 3). Although there appears to be a discontinuity in the proportion of those reporting zero health expenditure within formal private health facilities (Fig. 5a), this effect is statistically insignificant (Table 3). This is unsurprising given the high variance observed in the data points, particular above the eligibility threshold (Fig. 5a). Overall, for all those utilising formal private or informal health care services there appears to be no difference between both groups in the level of total health expenditure.

**Table 3:** The impact of the FHCI in formal and informal health facilities

	Formal private			Informal private/others		
	Simple linear	Linear interaction	Quadratic interaction	Simple linear	Linear interaction	Quadratic interaction
Utilisation (LPM)						
ITT effect (Age ≥ 5 years)	−0.0286	−0.0377	−0.0875	0.0995^**^	0.105^**^	0.0526
	(0.031)	(0.033)	(0.069)	(0.050)	(0.050)	(0.083)
*N*	1633	1633	1633	1633	1633	1633
*AIC*	566.7^a^	567.4	568.2	1774.2^a^	1776.0	1779.3
Probability of reporting zero consultation and prescription expenditure (LPM)						
ITT effect (Age ≥ 5 years)	0.0946	0.0980	0.0902	0.00674	−0.0694	−0.0659
	(0.197)	(0.193)	(0.339)	(0.081)	(0.094)	(0.146)
*N*	152	152	152	465	465	465
*AIC*	226.0^a^	228.0	231.9	519.6	518.6^a^	522.3
Total expenditure (two-part model showing marginal effects) ^b^						
ITT effect (Age ≥ 5 years)	−0.948	−1.104	−139.1	0.190	0.245	−3.891
	(1.239)	(1.525)	(308.367)	(0.201)	(0.194)	(10.624)
*N*	152	152	152	465	465	465
*AIC*	626.9	624.5	618.4^a^	419.6	419.1	417.1^a^

Control variables includes region dummies, head of households’ age and education

Standard errors in parentheses

*LPM* linear probability model

^**^ *p* < 0.05

^a^Optimal model specification

^b^Regional dummies excluded for informal private/others due to no observations in the Western region

### Impact on maternal health seeking behaviours

Approximately two thirds of the sample gave birth after the FHCI was in place and so were assumed to be affected by the policy (Table 4). The mean values of most covariates are similar for observations in the pre- and post-FHCI period although the proportion with secondary education is higher in the post-FHCI period. The majority of study outcomes are higher in the post-FHCI group. Of particular note is the increase in proportion receiving timely postnatal care (by 14.5 percentage points). Delivery with a skilled health worker and delivery at a government facility are 5.2 and 5.4 percentage points, respectively, higher in the post-FHCI period.

**Table 4:** Mean characteristics of women before and after the FHCI

Variables	Pre-FHCI	Post-FHCI	Difference (Post-Pre)	All
ANC Outcome Variables^a^				
Received skilled ANC during pregnancy	0.963	0.973	0.01	0.968
ANC received at public facility	0.955	0.969	0.014	0.964
Four ANC visits at public facility	0.751	0.771	0.02	0.508
*Obs.*	*2685*	*5630*		*8315*
PNC Outcome Variables				
Health Facility for Delivery				
Government facility	0.447	0.501	0.054	0.479
Government hospital	0.12	0.134	0.014	0.129
Government health centre	0.329	0.369	0.04	0.352
Private facility	0.017	0.018	0.001	0.017
Delivery with skilled health worker	0.584	0.636	0.052	0.616
Postnatal care within 48 h of birth in public facility	0.121	0.266	0.145	0.208
Vitamin A given within 2 months of delivery	0.816	0.788	−0.028	0.813
3 courses of DPT received within 12 months of delivery (DPT3) ^b^	0.609	0.625	0.016	0.691
*Obs.*	*3968*	*7970*		*11,938*
Covariates				
Age	30.5	28.38	−2.12	29.34
Urban	0.315	0.302	−0.013	0.308
Christian	0.19	0.187	−0.003	0.19
Mother’s education				
Primary education	0.127	0.142	0.015	0.133
Secondary education	0.136	0.184	0.048	0.164
Higher education	0.016	0.01	−0.006	0.012
Asset index quintile				
Lowest	0.232	0.232	0	0.233
Second	0.194	0.2	0.006	0.199
Middle	0.204	0.202	−0.002	0.199
Fourth	0.215	0.22	0.005	0.218
Highest	0.155	0.147	−0.008	0.15
Eastern Region	0.228	0.213	−0.015	0.221
Northern Region	0.384	0.396	0.012	0.385
Southern Region	0.279	0.277	−0.002	0.281
Western Region	0.11	0.114	0.004	0.113

^a^Policy implementation assumed to be 9 months prior to delivery date

^b^Deliveries excluded from November 2012 to allow for 1 year vaccination period

The regression models are estimated for two structural ‘breakpoints’. The first structural breakpoint is based on the official start of the policy. Given that the FHCI was introduced at the end of April 2010, a break point of May 2010 is used. The second is based on the rolling tobit estimation. Visual inspection and the use of the structural break function find a shift in the intercept for key variables around 2 months after policy implementation.^7^ Finding a lagged effect is perhaps not surprising since it is likely to take time for health care seeking behaviour to change. Both models are estimated for both policy breakpoints.

A simple before and after comparison, based on equation (4) suggests a statistically significant increase (*p* < 0.01) in ANC4, public facility deliveries, delivery with skilled health workers, postnatal care and 3^rd^ course DPT in the post-FHCI period (Table 5, Model 1). Similar changes in the proportion of women reporting each outcome (marginal effects) are reported for the two breakpoints. As expected, given that the FHCI mostly only applies to public services, there is no significant increase in private delivery care.

**Table 5:** Impact of the FHCI on the utilisation of maternal health services

	Model 1 (M1)		Model 2 (M2)		LL test (M2 vs. M1)
	May’10	June’10^a^	May’10	June’10	
Antenatal care (ANC) visits in public facilities^b^					
*Coefficients*					
Time trend			0.016	0.019^*^	
Policy	0.282^**^	0.245^*^	1.066^**^	1.059^*^	
Policy * Time trend			−0.026^*^	−0.029^*^	
*Marginal effects (%)*					
Policy	1.0^*^	0.8^*^	4.2	4.1	3.38
Four ANC visits in public facilities (ANC4)^b^					
*Coefficients*					
Time trend			0.004	0.003	
Policy	0.118^*^	0.125^**^	0.260	0.396	
Policy * Time trend			−0.005	−0.007	
*Marginal effects (%)*					
Policy	2.0^*^	2.2^**^	4.6	7.0	0.7
Delivery in public facility					
*Coefficients*					
Time trend			0.012^*^	0.006	
Policy	0.231^***^	0.295^***^	0.208^*^	0.330^**^	
Policy * Time trend			−0.007	−0.005	
*Marginal effects (%)*					
Policy	4.8^***^	6.1^***^	4.3^*^	6.8^**^	9.34
Delivery in private facility^c^					
*Coefficients*					
Time trend			0.002	0.009	
Policy	0.081	0.081	−0.236	−0.410	
Policy * Time trend			0.007	0.005	
Delivery with trained health worker^d^					
*Coefficients*					
Time trend			0.014^**^	0.005	
Policy	0.241^***^	0.310^***^	0.135	0.263^*^	
Policy * Time trend			−0.007	−0.002	
*Marginal effects (%)*					
Policy	4.6^***^	6.0^***^	2.6	5.1^*^	16.87
Postnatal care within 48 h					
*Coefficients*					
Time trend			0.033^***^	0.034^***^	
Policy	1.046^***^	1.072^***^	0.635^***^	0.814^***^	
Policy * Time trend			−0.013	−0.017^**^	
*Marginal effects (%)*					
Policy	14.1^***^	15.2^***^	9.1^***^	11.7^***^	89.8
Vitamin A given postnatally					
*Coefficients*					
Time trend			0.002	0.002	
Policy	0.035	−0.081	0.782^***^	0.924^***^	
Policy * trend			−0.022^*^	−0.024^**^	
*Marginal effects (%)*					
Policy	0.5	−1.2	13.2^***^	15.6^***^	116
Infant DPT full course^e, f^					
*Coefficients*					
Time trend			0.026^***^	0.026^***^	
Policy	0.670^***^	0.671^***^	0.536^***^	0.727^***^	
Policy * Time trend			−0.014	−0.019^**^	
*Marginal effects (%)*					
Policy	13.4^***^	13.3^***^	11.0^***^	14.0^***^	574

All models control for covariates listed in Table 4 & estimated with district and time (month) random effects

^*^ *p* < 0.1, ^**^ *p* < 0.05, ^***^ *p* < 0.01

^a^Alternative policy impact date suggested by the breakpoint function utilised

^b^Policy implementation assumed to be 9 months prior to delivery date

^c^Marginal effects could not be estimated

^d^Includes doctor, midwife and MCH aide

^e^Slightly lower but still significant result recorded where mother reported vaccinations also included

^f^Deliveries excluded from November 2012 to allow for 1 year vaccination period

Incorporating month and interaction terms into the model helps to separate the effect of the intervention from general trends in outcome variables (Table 5, Model 2). The marginal effects of the immediate policy impact are mostly larger in Model 2 compared to Model 1 particularly for services provided postnatally: 15.6 % for vitamin A supplementation (*p* < 0.01) and 14.0 % for full course DPT vaccination for infants (*p* < 0.01). The modest increase in delivery with a trained health worker (5.1 %, *p* < 0.1) contrasts with the larger impact (6.8 %, *p* < 0.05) for delivery in a government facility suggesting that some of the effect of the policy may be to incentivise women to deliver in a facility rather than with a trained health worker at home. The effects are generally larger for the breakpoint of June rather than May, supporting a lagged effect of the policy change. Likelihood ratio tests justify selection of Model 2 over Model 1 for all but use of private facilities and ANC4.

A general improvement in outcomes over time, independent of the policy, is suggested for delivery with a trained health worker, use of postnatal care and full course DPT to infants. The negative coefficients on most of the policy-time trend interactions suggest that the policy effect may diminish over time. In most cases this coefficient is lower in absolute value than the general trend term suggesting that outcomes continue to improve but at a slower rate than in the past. The results are robust to disaggregation by region and into rural and urban areas (the impact is slightly larger in rural areas).

## Discussion

This study investigates the extent to which Sierra Leone’s FHCI increased access to health care services and reduced financial risks associated with seeking health care among beneficiaries of the policy. Our results suggest that children under the age of 5 years (eligible children), are less likely to pay consultation and prescription charges in public health facilities where the FHCI applies. However this effect fails to translate into a discernible impact on children’s use of health care services. One reason why the FHCI may have had less impact than expected on utilisation is the costs that are still incurred by users. Although our findings show that eligible children are on average less likely to incur any out-of-pocket health expenditure in public facilities, a proportion of eligible children still do at levels comparable to non-eligible children. These findings are consistent with other studies which have shown that other beneficiaries of the FHCI (pregnant women and recent mothers), often bear the cost of medicines and medical supplies in public health care facilities [14, 15, 19]. In public health facilities, medicines are available either as FHCI drugs for FHCI beneficiaries or as cost recovery drugs for non-beneficiaries. Apparent shortages of FHCI medicines in these facilities often result in beneficiaries paying for medicines under the cost recovery scheme [14, 15]. The limited impact of the FHCI in removing or reducing financial barriers to accessing care may therefore explain the limited impact observed with the utilisation of public health facilities.

Additional factors that may be impeding greater impact include the remaining costs of accessing ‘free’ care, such as transportation costs which can be significant and sometimes more significant than user fees themselves [20]; and the extent to which human resource factors are successfully addressed and support positive staff attitudes to patients claiming exemptions and constrain the denial of exemptions or emergence of informal charges [21].

As expected, no effect is observed with the use of formal private care facilities but interestingly, we observe a significantly higher use of informal health care services among non-eligible children. This has important implications for the health of non-eligible children who appear to be seeking less expensive and potentially lower quality care from informal health providers and vendors. The absence of an effect on the probability of paying for health care services (and with the level of payments made) is expected given that the FHCI does not apply in informal health facilities.

For maternal care, the before- after analysis adjusted for time-trends suggests that the use of public facilities for delivery and postnatal care by women as well as for vaccination of infants increased in the post-FHCI period. The improvement is greater if a lagged effect (2 months) of policy is assumed. The policy changes are statistically significant and substantial but the impact seems to degrade over time, reflected in the negative interaction terms that reduce the pre-policy trend improvements in outcomes. These findings are supported by other studies which showed that the positive effect of the FHCI on utilisation observed within the first few months of implementation was not sustained [4, 8].

While not a focus of this study, it is worth noting that the improvements observed in the use of maternal health services may not necessarily translate into better health outcomes for mothers and children. For example, between the 2008 and 2013 DHS, no significant change was observed in infant and under-five mortality. Maternal mortality on the other hand appeared to have increased (although not statistically significant). Nevertheless in 2013, Sierra Leone ranked highest globally in terms of maternal mortality ratio [22].

Limitations of this study relate to methods and the time period for which data are available. The policy was introduced at a single time point for the whole country, which did not allow for control groups suitable for comparing with those affected. For the analysis of maternal health-seeking behaviour, the lack of a control group means that we cannot compare changes in the intervention group with a comparable counterfactual. We mitigate this to some extent by examining the effect on service use in the months before and after the implementation of the policy and incorporate a monthly time-trend into the model. We cannot be sure, however, that a similar change would not have occurred in a counterfactual group although it does seem unlikely that the same monthly changes would have been observed unless a separate policy with similar expected effects had been implemented at the same time. We are not aware of such a policy.

For the effect on children, the RDD approach permits the identification of effect using one time period. However, we are unable to verify causality of effect [23]. Instead an intention- to-treat effect is estimated due to lack of direct information on whether children were completely exempted from paying any fees in public health facilities. In this study receipt of free health care was inferred from responses to two survey questions – the use of health care services and consultation and prescription expenditure incurred as a result. We assume that children received free health care if no consultation and prescription expenditure was reported following a visit to a health facility. However, we cannot rule out payments made for the use of other health services such as diagnostic services. Furthermore, frequent medicine and medical supply shortages and stock-outs within health facilities [14, 15, 19] may result in ‘zero’ prescription expenditure being reported not because medication was received free of charge but because prescribed medicines were not available. A future household survey might aim to include a question about the exemption experience of children and mothers to allow identification of causal effects. Furthermore, the RDD is undertaken using data that were collected relatively soon after policy implementation. It may be that as the policy becomes better known and embedded in the system, the effect may increase. The declining effect on maternal service use is salutary, however, suggesting if anything, a reducing impact over time. Of course, in the period in which it might have settled and stabilised, the Ebola outbreak disrupted every aspect of health care provision in the country and therefore the availability of further data for later periods will not be able to settle this question.

## Conclusion

As with other cases of policy that focuses on relieving demand side constraints to health service use, impact is constrained by supply side capacity [21, 24]. In Sierra Leone’s case, a number of measures were taken to strengthen supply-side capacity, especially in relation to human resources [7, 21]. While some progress appears to have been made with the health workforce [7], the evidence suggests that supply side reforms were insufficient [21], perhaps especially in relation to ensuring sufficient medicines supply to support those receiving services under the FHCI. It is possible that the insignificant apparent effect of the FHCI on out-of-pocket expenditures made on behalf of children reflects weak monitoring and accountability within the medicines procurement and supply chain system [14, 15, 19]. Problems of service under-utilisation cannot be resolved by addressing the demand-side constraints only and supply-side constraints will have to be sufficiently addressed before the full potential of the FHCI can be realised. For example, governance and accountability within health facilities might be reinforced through the use of payment mechanisms (such as payment for performance) that explicitly reimburse health facilities for providing services to beneficiaries of the FHCI [25]. Other supplementary demand-side policies that address physical barriers to accessing health facilities are equally germane particularly in rural areas where health facilities are sparsely distributed and accessibility made worse by poor road networks [26].

The 2014–15 Ebola out-break in Sierra Leone has cast the failings of its health system into sharp relief, but may also be the harbinger of new resources and commitment to health system strengthening, through which a reinforced FHCI might achieve its undoubted potential to improve women and children’s access to health care.

## Appendix 1

**Table 6:** Impact of the FHCI in Public/NGO/Missionary Health Facilities by Household Location

	Rural			Urban		
	Simple linear	Linear interaction	Quadratic interaction	Simple linear	Linear interaction	Quadratic interaction
Utilisation (LPM)						
ITT effect (Age ≥ 5 years)	−0.0614	−0.0534	−0.0516	−0.0243	−0.0039	0.265
	(0.057)	(0.063)	(0.102)	(0.124)	(0.120)	(0.195)
*N*	*1200*	*1200*	*1200*	*433*	*433*	*433*
*AIC*	*1554.1* ^*a*^	*1555.9*	*1559.5*	*587.2*	*587.7*	*581.8* ^*a*^
ITT effect (Age ≥ 5 years)	−0.136^*^	−0.143^**^	−0.162	−0.159	−0.169	0.113
	(0.073)	(0.072)	(0.119)	(0.173)	(0.174)	(0.256)
*N*	*707*	*707*	*707*	*208*	*208*	*208*
*AIC*	*900.3* ^*a*^	*902.0*	*905.9*	*283.3* ^*a*^	*285.0*	*285.6*
ITT effect (Age ≥ 5 years)	−0.210	−0.276	0.563	0.709	0.517	−4.024
	(0.424)	(0.508)	(1.302)	(1.165)	(0.915)	(4.105)
*N*	*707*	*707*	*707*	*208*	*208*	*208*
*AIC*	*586.2*	*585.9*	*585.6* ^*a*^	*878.4*	*876.2*	*875.7* ^*a*^

Control variables includes region dummies, head of households’ age and education

Standard errors in parentheses

*LPM* linear probability model

^*^ *p* < 0.1, ^**^ *p* < 0.05

^a^Optimal model specification

## Appendix 2

**Table 7:** Impact of the FHCI in Public/NGO/Missionary Health Facilities by Total Household Expenditure

	Below median expenditure			Above median expenditure		
	Simple linear	Linear interaction	Quadratic interaction	Simple linear	Linear interaction	Quadratic interaction
Utilisation (LPM)						
ITT effect (Age ≥ 5 years)	0.0394	0.0495	0.113	−0.0843	−0.0702	0.130
	(0.073)	(0.077)	(0.118)	(0.078)	(0.076)	(0.155)
*N*	*799*	*799*	*799*	*834*	*834*	*834*
*AIC*	*1092.1* ^*a*^	*1093.7*	*1094.6*	*1115.6*	*1117.1*	*1115.6*
ITT effect (Age ≥ 5 years)	−0.125	−0.151^*^	−0.00640	−0.149	−0.140	−0.149
	(0.090)	(0.090)	(0.141)	(0.097)	(0.098)	(0.170)
*N*	*445*	*445*	*445*	*470*	*470*	*470*
*AIC*	*578.1*	*577.7* ^*a*^	*580.4*	*607.8* ^*a*^	*609.5*	*613.2*
ITT effect (Age ≥ 5 years)	0.413	0.406	0.866	−0.0412	−0.0249	−2.412
	(0.499)	(0.398)	(1.876)	(0.603)	(0.585)	(4.017)
*N*	*445*	*445*	*445*	*470*	*470*	*470*
*AIC*	*615.1*	*614.4*	*613.0* ^*a*^	*705.0*	*704.7*	*703.6* ^*a*^

Control variables includes region dummies, head of households’ age and education

Standard errors in parentheses

*LPM* linear probability model

^*^ *p* < 0.1

^a^Optimal model specification

## Footnotes

1 These include traditional healers, traditional birth attendants, spiritualist, patent medicine vendors and other health workers in undefined facilities.

2 Health care expenditure was originally collected in Leones. We converted this to USD using 2011 exchange rate.

3 Here, a binary variable that describes beneficiaries of the FHCI captures the treatment effect as the difference in outcomes between beneficiaries and non-beneficiaries.

4 Free health care is inferred from reporting visits to a health care facility and zero consultation and prescription expenditure.

5 Model specification yielding the lowest AIC is considered the optimal specification that best fits the data.

6 Figures 4, 5, 6 are estimated similarly to Figs. 1, 2, 3.

7 For delivery with a skilled health worker, the function estimates a breakpoint in June 2010, Wald test *p* < 0.001.
